# Guillain-Barré Syndrome and Healthcare Needs during Zika Virus Transmission, Puerto Rico, 2016

**DOI:** 10.3201/eid2301.161290

**Published:** 2017-01

**Authors:** Emilio Dirlikov, Krista Kniss, Chelsea Major, Dana Thomas, Cesar A. Virgen, Marrielle Mayshack, Jason Asher, Luis Mier-y-Teran-Romero, Jorge L. Salinas, Daniel M. Pastula, Tyler M. Sharp, James Sejvar, Michael A. Johansson, Brenda Rivera-Garcia

**Affiliations:** Centers for Disease Control and Prevention, Atlanta, Georgia, USA (E. Dirlikov, K. Kniss, C. Major, D. Thomas, M. Mayshack, L. Mier-y-Teran-Romero, J.L. Salinas, D.M. Pastula, T.M. Sharp, J. Sejvar, M.A. Johansson);; Puerto Rico Department of Health, San Juan, Puerto Rico (E. Dirlikov, D. Thomas, M. Mayshack, B. Rivera-Garcia);; University of California, San Diego, California, USA (C.A. Virgen);; Biomedical Advanced Research and Development Authority, Washington, DC, USA (J. Asher);; University of Colorado, Denver, Colorado, USA (D.M. Pastula)

**Keywords:** Zika virus, Guillain-Barré syndrome, Puerto Rico, surveillance, immunoglobulin, viruses, vector-borne infections

## Abstract

To assist with public health preparedness activities, we estimated the number of expected cases of Zika virus in Puerto Rico and associated healthcare needs. Estimated annual incidence is 3.2–5.1 times the baseline, and long-term care needs are predicted to be 3–5 times greater than in years with no Zika virus.

Guillain-Barré syndrome (GBS) is an autoimmune disorder characterized by varying degrees of weakness, sensory abnormalities, and autonomic dysfunction due to peripheral nerve or nerve root damage (*1*). Annual GBS incidence worldwide is ≈1.1–1.8 cases/100,000 population and varies by geography and age group (*2*,*3*). Death is rare and is usually caused by respiratory failure, autonomic dysfunction, or deep vein thrombosis (*4*).

GBS has been associated with various infectious agents, including Zika virus (*5*). Zika virus is a flavivirus transmitted primarily by *Aedes* species mosquitoes; symptoms of infection include rash, arthralgia, and fever (*6*). During a 2013–2014 outbreak in French Polynesia, 42 cases of GBS were reported during a 7-month period, compared with 3–10 cases annually in previous years; all GBS patients during the outbreak had Zika virus antibodies (*7*).

In December 2015, the Puerto Rico Department of Health reported local transmission of Zika virus (*8*). In February 2016, the Department of Health reported the first case of Zika virus–associated GBS and established the GBS Passive Surveillance System, with support from the Centers for Disease Control and Prevention (*9*). During January 1–July 31, 2016, a total of 56 cases of GBS were reported; evidence of Zika virus or flavivirus infection was found in 34 (61%) of these (*9*). As in other locations (*5*), GBS cases in Puerto Rico are anticipated to increase with ongoing Zika virus transmission. To assist with public health preparedness activities, we estimated the annual number of expected cases of GBS and associated healthcare needs in Puerto Rico (Technical Appendix).

## The Study

We estimated the weekly number of cases of GBS and associated healthcare needs for 3 scenarios: 1) in the absence of Zika virus transmission; 2) in an average week during Zika virus transmission; and 3) during the peak week of Zika virus transmission (Table). Estimates were derived from baseline and Zika virus–associated GBS cases. The population of Puerto Rico in 2015 was estimated at 3,474,182 persons (*10*).

**Table:** Estimates of weekly Guillain-Barré syndrome cases and healthcare resource needs, Puerto Rico, 2016

Variable	Scenario*	Estimate		
		Median	Interquartile range	95% Uncertainty interval
New cases and long-term care patients				
Case-patients	No Zika virus	1	0–2	0–4
	Average week during Zika virus	5	3–6	1–11
	Peak week during Zika virus	11	6–17	1–34
Long-term care patients	No Zika virus	0	0–1	0–2
	Average week during Zika virus	2	1–3	0–6
	Peak week during Zika virus	5	2–8	0–16
New patient healthcare resource needs				
Intravenous immunoglobulin	No Zika virus	1	0–2	0–3
	Average week during Zika virus	4	3–6	0–10
	Peak week during Zika virus	10	5–15	0–30
Mechanical ventilation	No Zika virus	0	0–0	0–1
	Average week during Zika virus	1	0–2	0–3
	Peak week during Zika virus	2	1–4	0–8
Regular ward beds	No Zika virus	0	0–1	0–3
	Average week during Zika virus	3	1–4	0–7
	Peak week during Zika virus	6	3–10	0–21
Intensive care unit beds	No Zika virus	0	0–1	0–2
	Average week during Zika virus	2	1–3	0–6
	Peak week during Zika virus	4	2–7	0–15

*The weekly number of Guillain-Barré syndrome cases and associated healthcare needs were estimated for 3 scenarios: 1) in the absence of Zika virus transmission; 2) in an average week during Zika virus transmission; and 3) during the peak week of Zika virus transmission.

We calculated baseline GBS incidence in Puerto Rico by using data collected through medical chart review of patients suspected to have GBS at 9 reference hospitals in Puerto Rico during 2012–2015 and for whom neurologic diagnosis was confirmed by the Brighton Collaboration criteria (*11*). The 2013 incidence of GBS was 1.7 cases (95% CI 1.3–2.1 cases) per 100,000 population (J.L. Salinas, unpub. data). Using this incidence range, in the absence of Zika virus transmission, we estimated that 1 (interquartile range [IQR] 0–2) case occurs each week, and 59 (IQR 52–66) cases occur each year.

We assumed that, during Zika virus transmission, ≈25% of the population could have been infected during 2016, similar to recent chikungunya and dengue virus epidemics in Puerto Rico (*12*). We used a triangular distribution to characterize uncertainty, with a minimum estimate of 10% infected and a maximum estimate of 70% infected (*12*,*13*). Estimated GBS risk associated with Zika virus infection was assumed to be 1.1–2.3 cases/10,000 infections on the basis of a separate analysis of data aggregated from French Polynesia (*7*), Yap (*13*), Brazil (*14*), Colombia, El Salvador, Honduras, the Dominican Republic, and Puerto Rico (L. Mier-y-Teran, unpub. data). We used Monte Carlo sampling to draw 1 million values from each of these distributions to estimate each outcome of interest and its associated uncertainty.

We estimated that in an average week of Zika virus transmission, ≈5 (IQR 3–6) GBS cases would occur, comprising cases associated with baseline risk and Zika virus infection. As in previous outbreaks of Zika virus and other arboviral diseases, peak weekly incidence could be 2–4 times higher than average incidence. A maximum of 11 (IQR 6–17) cases could occur during the peak week, 2–4 times more than the average number of cases. We predicted that ≈241 (IQR 191–305) cases would occur in 2016.

Assumptions regarding treatment needs were also based on data collected from the 2012–2015 Puerto Rico hospitalized patient chart review (J.L. Salinas, unpub. data). We made the following assumptions: 90% of patients will require treatment with intravenous immunoglobulin (IVIg), 20% will require mechanical ventilation, all patients will be hospitalized, 40% will require intensive care, and 45% will require long-term care.

In the absence of Zika virus transmission, the estimated weekly number of new patients treated with IVIg was 1 (IQR 0–2) patient and of those requiring mechanical ventilation was low (IQR 0–0 patients). Estimates for the weekly number of new patients requiring a regular or intensive care unit (ICU) bed were also low (IQR 0–1 patient).

During an average week of Zika virus transmission, ≈4 (IQR 3–6) new patients would need treatment with IVIg, and 1 (IQR 0–2) new patient would require mechanical ventilation. An estimated 3 (IQR 1–4) new patients would require a regular ward bed, whereas 2 (IQR 1–3) new patients would require an ICU bed. During the peak week, ≈10 (IQR 5–15) new patients would need treatment with IVIg, and 2 (IQR 1–4) new patients would require mechanical ventilation. An estimated 6 (IQR 3–10) new patients would require a regular ward bed, whereas 4 (IQR 2–7) new patients would require an ICU bed.

An estimated 0 (IQR 0–1) patients would require long-term care during a week without Zika virus transmission, 2 (IQR 1–3) patients during an average week of Zika virus transmission, and 5 (IQR 2–8) patients during the peak week. During 2016, ≈108 (IQR 85–138) GBS patients would require long-term care.

## Conclusions

We estimated that there would be 191–305 new cases of GBS in Puerto Rico in 2016, comprising baseline and Zika virus–associated cases. This estimate represents an annual incidence of 5.5–8.7 cases/100,000 population, which is 3.2–5.1 times the baseline incidence. Associated healthcare resource needs will increase accordingly. Estimated long-term care needs in 2016 were predicted to be 3–5 times greater than in years with no Zika virus transmission.

These estimates have limitations. First, there is considerable uncertainty around key assumptions, including that increases in GBS incidence will mirror those experienced in other Zika virus–affected countries (*5*,*7*,*13*,*14*). Second, the estimates of associated healthcare needs did not address all possible needs, such as alternative treatments (i.e., plasmapheresis) or additional treatments, such as those for neuropathic pain, cardiac arrhythmias, and deep vein thrombosis. Third, estimates assumed 1 peak week, although GBS cases tend to cluster, and multiple peaks could occur. Finally, a causal association between Zika virus infection and GBS has not been definitively established.

Continued GBS surveillance will monitor for increased incidence and enable adapted public health response. Healthcare workers, including internists, family physicians, and nurses, might need training to ensure adequate patient clinical management if GBS cases increase as predicted. The Puerto Rico Department of Health and the Centers for Disease Control and Prevention have developed training material toward this end. The availability and accessibility of GBS treatment, especially IVIg, and long-term care services should be evaluated, especially given the high costs of GBS patient care (*15*). The Puerto Rico Department of Health is also creating an inventory of available and expandable resources, working with manufacturers and distributors to understand supply chains, and facilitating prompt treatment delivery at points of care.

Technical Appendix Detailed methods and parameters used in the model for a study of Guillain-Barré syndrome and healthcare needs during Zika virus transmission, Puerto Rico, 2016.
