# Supplementary material for: Guillain-Barré Syndrome and Healthcare Needs during Zika Virus Transmission, Puerto Rico, 2016
Source: Emerg Infect Dis. 2017 Jan;23(1):134–6. doi: 10.3201/eid2301.161290 (PMC5176211; doi:10.3201/eid2301.161290)
Supplement: Technical Appendix — Detailed methods and parameters used in the model for a study of Guillain-Barré syndrome and healthcare needs during Zika virus transmission, Puerto Rico, 2016. [file 16-1290-Techapp-s1.pdf]

Article DOI: http://dx.doi.org/10.3201/eid2301.161290

Guillain-Barré Syndrome and Healthcare Needs during Zika Virus Transmission, Puerto Rico, 2016

Technical Appendix

Methods

We used Monte Carlo sampling with 1 million realizations to estimate case numbers and resources required. Each realization drew a value of the Guillain-Barré syndrome (GBS) baseline risk, the risk for Zika virus infection, the risk of GBS after Zika virus infection, and the relative incidence at the peak compared with an average week from the distributions specified in the previous section. Once these parameters were selected, the different components of our model were obtained as follows:

Total Number of Baseline and Zika Virus–Associated GBS Cases

The number of baseline GBS cases during the outbreak was sampled from a binomial process for the population of Puerto Rico with a probability equal to the baseline rate, assuming a time span of 1 year. The total number of Zika virus–associated GBS cases during the outbreak was sampled from a binomial process for the population of Puerto Rico with a probability equal to the product of the attack rate and the GBS risk after a Zika virus infection.

Number of Weekly Baseline GBS Cases in the Absence of Zika Virus

The mean number of weekly baseline GBS cases was sampled from a Poisson process with a mean equal to the total number of baseline GBS cases divided by 52, the number of weeks in a year.

Number of Zika Virus–Associated GBS Cases in an Average Week and Peak Week during Zika Virus Transmission

The mean number of weekly Zika virus–associated GBS cases was sampled from a Poisson process with a mean equal to the total number of Zika virus–associated cases divided by

the outbreak duration in weeks (52, the assumed length of the epidemic). The mean number of weekly Zika virus–associated GBS cases in the peak week was sampled by using a Poisson process. The mean was equal to the average number of Zika virus–associated cases per week multiplied by the relative sampled incidence at peak compared with an average week.

Resource Needs for GBS Patients

For each weekly estimate of cases, we estimated the proportion requiring different levels of healthcare from a binomial process with the respective probability (Technical Appendix Table) and the number of estimated cases.

Code

Estimates were generated using R (*1*). The following code was used:

```
rm(list = ls())

require(data.table)

require(dplyr)

require(triangle)

set.seed(8675309)

n_samples <- 1e6

### fixed parameters

PR_population <- 3474182

GBS_long_term_care_fraction <- 0.45

GBS_IVIG_fraction <- 0.9

GBS_novent_ICU_fraction <- 0.2

GBS_vent_ICU_fraction <- 0.2

GBS_hosp_nonICU_fraction <- 0.6

zika_outbreak_duration_weeks <- 52

### simulation table

sim.data <- data.table(
```

```
GBS_baseline_rate = rnorm(n_samples, 1.7e-5, 0.2e-5),
zika_attack_rate = rtriangle(n_samples, 0.1, 0.7, 0.25),
GBS_zika_rate = rnorm(n_samples, 1.6e-4, 0.31e-4),
peak_mean_weekly_incidence_ratio = runif(n_samples, 2, 4)
) %>%
mutate(
GBS_total_background_cases =
rbinom(n_samples, PR_population, GBS_baseline_rate),
GBS_weekly_background_cases_mean =
rpois(n_samples, GBS_total_background_cases / zika_outbreak_duration_weeks),
GBS_total_zika_cases =
rbinom(n_samples, PR_population, zika_attack_rate * GBS_zika_rate),
GBS_weekly_zika_cases_mean =
rpois(n_samples, GBS_total_zika_cases / zika_outbreak_duration_weeks),
GBS_total_cases = GBS_total_zika_cases + GBS_total_background_cases,
GBS_weekly_cases_mean = GBS_weekly_zika_cases_mean + GBS_weekly_background_cases_mean,
GBS_weekly_cases_peak = GBS_weekly_background_cases_mean +
rpois(n_samples, GBS_weekly_zika_cases_mean * peak_mean_weekly_incidence_ratio),
GBS_total_cases_long_term = rbinom(n_samples, GBS_total_cases, GBS_long_term_care_fraction),
GBS_total_cases_background_long_term = rbinom(n_samples, GBS_total_background_cases,
GBS_long_term_care_fraction)
```

```
)
get_resource_needs = function(GBS_weekly_cases) {
output = rbind(
data.table(Resource = “Cases per Week,”
Value = GBS_weekly_cases),
data.table(Resource = “Long-Term Care Patients,”
Value = rbinom(n_samples, GBS_weekly_cases, GBS_long_term_care_fraction)),
data.table(Resource = “Ventilators,”
Value = rbinom(n_samples, GBS_weekly_cases, GBS_vent_ICU_fraction)),
data.table(Resource = “IVIg per Week,”
Value = rbinom(n_samples, GBS_weekly_cases, GBS_IVIG_fraction)),
data.table(Resource = “Non-ICU Beds,”
Value = rbinom(n_samples, GBS_weekly_cases, GBS_hosp_nonICU_fraction)),
data.table(Resource = “ICU Beds,”
Value = rbinom(n_samples, GBS_weekly_cases,
GBS_novent_ICU_fraction + GBS_vent_ICU_fraction)))
return(output)
}
raw_weekly_resources <- rbind(
cbind(get_resource_needs(sim.data$GBS_weekly_background_cases_mean),
Estimate = “No Zika”),
cbind(get_resource_needs(sim.data$GBS_weekly_cases_mean),
Estimate = “Average Zika”),
cbind(get_resource_needs(sim.data$GBS_weekly_cases_peak),
```

```
Estimate = “Peak Zika”)) %>%
mutate(
Estimate = factor(Estimate, c(“No Zika,” “Average Zika,” “Peak Zika”)),
Resource = factor(Resource, c(“Cases per Week,” “Long-Term Care Patients,”
“IVIg per Week,” “Ventilators,” “Non-ICU Beds,” “ICU Beds”)))
### summarize
summarized_data <- raw_weekly_resources %>%
as.data.frame() %>%
group_by(Resource, Estimate) %>%
do({
quantile_frame =
data.frame(t(quantile(.$Value, probs = c(0.025, 0.25, 0.5, 0.75, 0.975))))
colnames(quantile_frame) = c(“Low,” “IQR_Low,” “Mid,” “IQR_High,” “High”)
return(quantile_frame)
}) %>%
mutate(
Median = signif(Mid, 2),
IQR = paste0(signif(IQR_Low, 2), “–,” signif(IQR_High, 2)),
`95% UI` = paste0(signif(Low, 2), “–,” signif(High, 2))) %>%
select(Resource, Estimate, Median, IQR, `95% UI`) %>%
arrange(Resource, Estimate)
summarized_data[]
```

References

1. R Project. R: A language and environment for statistical computing. Vienna: R Foundation for Statistical Computing; 2016.

2. US Census Bureau. Puerto Rico commonwealth totals: vintage 2015 [cited 2016 May 9]. http://www.census.gov/popest/data/puerto_rico/totals/2015/index.html

3. Duffy MR, Chen TH, Hancock WT, Powers AM, Kool JL, Lanciotti RS, et al. Zika virus outbreak on Yap Island, Federated States of Micronesia. N Engl J Med. 2009;360:2536–43. http://dx.doi.org/10.1056/NEJMoa0805715

4. Duffy MR, Chen TH, Hancock WT, Powers AM, Kool JL, Lanciotti RS, et al. Zika virus outbreak on Yap Island, Federated States of Micronesia. N Engl J Med. 2009;360:2536–43. http://dx.doi.org/10.1056/NEJMoa0805715

5. Cardoso CW, Paploski IA, Kikuti M, Rodrigues MS, Silva MM, Campos GS, et al. Outbreak of exanthematous illness associated with Zika, chikungunya, and dengue viruses, Salvador, Brazil. Emerg Infect Dis. 2015;21:2274–6. http://dx.doi.org/10.3201/eid2112.151167

Technical Appendix Table. Parameters used in the model for a study of Guillain-Barré syndrome and healthcare needs during Zika virus transmission, Puerto Rico, 2016*

Parameter	Estimated value	Distribution	Source	Citation
Puerto Rico population, 2016	3,474,182	Fixed	US Census	US Census (*2*)
Baseline GBS rate	1.7/100,000 population/year	Gaussian, σ = 0.20	Chart review of Puerto Rico GBS patients	(J.L. Salinas, unpub. data)
Zika virus infection risk in Puerto Rico, 2016	25% peak probability	Triangle, min 10%, max 70%	Historic CHIKV and DENV epidemics in Puerto Rico	(*3*)
Fraction of Zika virus infections developing GBS	1.6/10,000 infections	Gaussian, σ = 0.31	Analysis of country-level GBS and Zika virus reports	(L. Mier-y-Teran, unpub. data) (*3–5*)
Relative Zika virus incidence during peak week compared with an average week	2–4 times	Uniform	Analysis of country-level GBS and Zika virus reports	(L. Mier-y-Teran, unpub. data)
Percentage of GBS patients needing long-term care	45	Fixed	Chart review of Puerto Rico GBS patients	(J.L. Salinas, unpub. data)
Percentage of GBS patients needing IVIg	90	Fixed	Chart review of Puerto Rico GBS patients	(J.L. Salinas, unpub. data)
Percentage of GBS patients needing an ICU bed	40	Fixed	Chart review of Puerto Rico GBS patients	(J.L. Salinas, unpub. data)
Percentage of GBS patients needing an ICU bed and a ventilator	20	Fixed	Chart review of Puerto Rico GBS patients	(J.L. Salinas, unpub. data)
Percentage of GBS patients needing an ICU bed but no ventilator	20	Fixed	Chart review of Puerto Rico GBS patients	(J.L. Salinas, unpub. data)
Percentage of GBS patients needing a regular ward bed	60	Fixed	Chart review of Puerto Rico GBS patients	(J.L. Salinas, unpub. data)

*CHIKV, chikungunya virus; DENV, dengue virus; GBS, Guillain-Barré syndrome; ICU, intensive care unit; IVIg, intravenous immunoglobulin.
